# Perceived effective and feasible strategies to promote healthy eating in young children: focus groups with parents, family child care providers and daycare assistants

**DOI:** 10.1186/s12889-016-3710-9

**Published:** 2016-10-04

**Authors:** Laura Vandeweghe, Ellen Moens, Caroline Braet, Wendy Van Lippevelde, Leentje Vervoort, Sandra Verbeken

**Affiliations:** 1 Department of Developmental, Personality and Social Psychology, Ghent University, Henri Dunantlaan 2, 9000 Ghent, Belgium; 2 Department of Public Health, Ghent University, De Pintelaan 185, 9000 Ghent, Belgium

**Keywords:** Young children, Healthy eating, Caregivers, Focus groups, Parents, Family child care providers, Daycare assistants, Strategies

## Abstract

**Background:**

The aim of the current study is to identify strategies to promote healthy eating in young children that can be applied by caregivers, based on their own perceptions of effectiveness and feasibility. Whereas previous research mainly focused on parental influences on children’s eating behavior, the growing role of other caregivers in the upbringing of children can no longer be denied.

**Methods:**

Four focus groups were conducted with three types of caregivers of post-weaning children under 6 years old: parents (*n* = 14), family child care providers (*n* = 9), and daycare assistants (*n* = 10). The audiotaped focus group discussions were transcribed and imported into Nvivo 10.0 for thematic analysis. The behaviors put forward by the caregivers were categorized within three broad dimensions: global influences, general behaviors, and specific feeding practices.

**Results:**

Perceived effective strategies to promote healthy eating behavior in children included rewards, verbal encouragement, a taste-rule, sensory sensations, involvement, variation, modeling, repeated exposure, and a peaceful atmosphere. Participants mainly disagreed on the perceived feasibility of each strategy, which largely depended on the characteristics of the caregiving setting (e.g. infrastructure, policy).

**Conclusions:**

Based on former research and the current results, an intervention to promote healthy eating behaviors in young children should be adapted to the caregiving setting or focus on specific feeding practices, since these involve simple behaviors that are not hindered by the limitations of the caregiving setting. Due to various misconceptions regarding health-promoting strategies, clear instructions about when and how to use these strategies are necessary.

**Electronic supplementary material:**

The online version of this article (doi:10.1186/s12889-016-3710-9) contains supplementary material, which is available to authorized users.

## Background

The increasing prevalence of overweight and obesity is a global problem. The most problematic region is the United States, where 16.9 % of children between 2 and 19 years old are obese [1]. Europe is heading in the same direction, with 7.1 % of all children aged 2 to 10 years being classified as obese [2]. Since being overweight in childhood tends to persist in adolescence and adulthood [3, 4], tackling its causes at a young age is critical and a healthy diet during the first years of life is of the utmost importance [5, 6]. The various vitamins and minerals found in fruit and vegetables, for example, are essential for age-adequate development and growth. Nevertheless, young children’s consumption of fruit and vegetables is too low and does not meet the recommended daily intake [7]. To address this problem in childcare [8, 9] as well as at home [10] various intervention programs and studies have been designed. While interventions in childcare seem to elicit positive changes in diet quality or policy, their translation into children’s actual eating behaviors (e.g. the consumption of healthy food) has not been consistently observed [8]. Furthermore, most studies so far have been conducted in the United States and their results cannot simply be generalized to other cultures. More research is thus needed on how strategies can be implemented to improve healthy eating behavior in other (European) childcare settings.

Past research has shown that healthy food consumption in young children may be hindered by food neophobia (i.e. the rejection of novel or unknown foods) [11], and picky or fussy eating (i.e. the rejection of familiar foods) [12], however, it is generally determined by food preferences [13, 14]. Therefore, a clear understanding of how food preferences are shaped is fundamental to increasing the consumption of healthy food. Our innate aversion to bitter and sour flavors [15, 16] and our innate preference for sweet flavors [16, 17] suggests that an appreciation of most vegetables is not intrinsic but should be learned in interaction with the primary caregivers. Although previous research has mainly focused on parental influence on children’s eating development, it is clear that *other caregivers* are increasingly playing a role in the upbringing of children. We live in a society where two working parents is the rule rather than the exception [18]. In Flanders, when both parents work fulltime, 86 % regularly make use of the child care system [19]. These statistics indicate that we cannot ignore the growing role played by child care providers, including family child care providers and daycare assistants, in the raising of young children. Family child care providers offer to care for children in their own private home and supervise a small group of children on their own. Daycare assistants provide childcare in daycare centers, where children are under supervision of several daycare assistants. If we aim to improve the eating behavior of young children, research on health improvement strategies should thus include all caregivers.

The influence of caregivers on children’s eating behavior can be organized into three categories, in line with the comprehensive model of Rhee [20], which describes three categories of *parental* influences: specific parent feeding practices, general parent behaviors, and global parenting influences. The first category, specific parent feeding practices, are defined as “targeted toward the child, with the intent to shape eating behaviors and intake” (p. 13) (e.g. prompting the child to eat, rewarding it, encouraging it). In contrast, the second category contains general parent behaviors that are not directly targeted toward the child, but still have an indirect influence on its eating behavior (e.g. exposing the child to food, making foods available, modeling behavior). Finally, the third category refers to global parenting influences, which include parenting style and family functioning. These are responsible for the emotional climate at home in which the two first categories of parental influences are expressed and interpreted by the child. While parenting style is defined as the general pattern of parenting, family functioning is a broader dimension representing how the family manages daily routines, connects with each other, communicates and fulfills parenting roles. The three broad dimensions are obviously interconnected; for example, children are more inclined to accept specific parent behaviors in a positive family climate, created by positive family interactions or order in the household [20]. Some influences are positive and create a healthy eating environment for children, while others are negative and may contribute to unhealthy eating habits and overweight [20].

Several interventions have been designed for parents [10] and other child care providers [8] to improve children’s diets. However, a well-known problem is that interventions often fail to reach the target population [21]. It has been postulated that, besides an effective strategy for behavioral change, interventions also need to address participants’ motivation to engage in the program [21]. After all, the success of the program hinges on this motivation, so it is imperative that the concerns and motivations of the stakeholders (i.e. the people who are involved in the intervention) are integrated into the intervention program [22]. Nevertheless, little research has been done on the perceptions, beliefs and attitudes of those who have to apply health-promoting behaviors. To fill this research gap, the aim of the current study is to gain more insight into the perceptions of caregivers in Flanders, more specifically what they believe to be *effective* and *feasible* strategies to promote healthy eating in young children. The caregivers’ perceptions will be discussed in the light of scientific research on how to improve children’s eating behavior. Based on this, an evidence-based intervention program for childcare settings could be developed that takes into account the perceptions of people in actual practice.

## Methods

The present qualitative study was conducted as part of the REWARD project. The overall aim of this project is to provide evidence for a new public health framework to improve the eating patterns of children and adolescents by using learning paradigms and focusing on individual differences in reward sensitivity. Strategies that are perceived as feasible and effective as well as scientifically recommended will be tested in a laboratory as well as in a natural setting. The project will also investigate whether the effectiveness of strategies depends on child characteristics. The current study examines caregivers’ perspectives (i.e. parents, family child care providers and daycare assistants) on the effectiveness and feasibility of strategies used to stimulate healthy eating in young children.

There were several reasons why focus group interviews were chosen as the method of data collection. Firstly, this allowed us to identify a wide range of feelings, beliefs and perspectives on the topic. Secondly, a group interview generates interaction and makes participants think about specific examples of strategies that would remain uncovered with other methods of data collection, such as questionnaires or individual interviews. This interaction also makes it much easier to avoid suggestive or leading questions that hint at a specific strategy. Finally, firsthand information and perceptions from people in actual practice (i.e. a bottom-up approach) are equally valuable as strategies that have been experimentally proven to be effective (i.e. a top-down approach).

After the focus groups, the input that was gathered from the caregivers was placed within the larger context of existing scientific research. Based on actual practice and science, an informed decision could then be made about which intervention strategies should be disseminated among caregivers.

### Participants

In total, 33 caregivers (i.e. parents, family child care providers and daycare assistants) of post-weaning children under 6 years old took part in this study. Descriptive statistics are depicted in Table 1.

**Table 1:** Descriptive statistics of participants

Type of caregiver	Age		Sex
	Mean	SD	
Daycare assistants (*n* = 10)	36.1	6.87	1 male / 9 female
Family child care providers (*n* = 9)	33.0	4.24	1 male / 8 female
Parents (*n* = 14)	31.5	4.32	1 male / 13 female

### Procedure

The participants were recruited by the Flemish Centre for Innovation in the Early Years (VBJK) through personal contacts. Since this research center is in close contact with a variety of caregivers, it was able to recruit a purposive sample and convince people who would normally not volunteer for such group discussions. Two focus groups consisted of parents (*n* = 7 for each focus group), one focus group of family child care providers (*n* = 9) and one focus group of daycare assistants (*n* = 10). The four focus groups were conducted in a comfortable and neutral room at VBJK between March and December 2013. They were led by two VBJK researchers who were experienced in conducting focus groups. The discussions lasted approximately 90 min and were audio-recorded instead of videotaped to encourage participants to speak openly. At the start of every focus group discussion, the participants signed an informed consent form. During the discussion, fruit, cookies, coffee and water were provided. Afterwards, participants were thanked and received €10. The study procedure was approved by the ethical commission of the Faculty of Psychology and Educational Sciences at Ghent University.

In order to obtain standardization and consistency, a semi-structured questioning guide, developed by VBJK and reviewed by researchers from Ghent University, was used to lead each focus group discussion. Given the different roles played by caregivers in children’s lives, some questions were adapted and tailored to the specific type of caregiver. The questioning guide included meal-relevant questions, which were related to events before the meal or events during the meal. For every new topic, the discussion began with a question about the state of affairs (see Table 2 for examples of questions used in each focus group discussion). This is a useful icebreaker as it does not require anyone to voice an opinion yet and therefore puts people at ease. As an added bonus, by being reminded of what things were like in their own setting, the participants could more easily respond to the more important questions about the effectiveness of particular strategies. Moreover, the state of affairs gave us an impression of their setting, which in turn allowed us to derive information about the feasibility of certain strategies. More details on the study method can be found in a completed COREQ checklist [23], which is attached as an Additional file 1.

**Table 2:** Examples of questions used in each focus group discussion

Domain	Questions
State of affairs	
Before the meal	-Tell me something about the menu. Who chooses the menu? What’s on the menu? -Tell me something about the preparation of the food. Who prepares the food? Where and how is it prepared?
During the meal	-What is the atmosphere like during the meal? Is it noisy? How many children sit at the table? -How is the food presented? How is the food offered? -Can you describe a typical meal? What is the policy concerning eating?
Strategies	What, do you think, causes a child to enjoy eating, to be curious about food, to be willing to taste?

Note. Given the different role of the caregivers in the lives of the children, some questions were adapted and specifically tailored to the type of caregiver (i.e. parents, family child care providers and daycare assistants)

### Data analysis

The audiotaped focus groups discussions were transcribed and imported into Nvivo 10.0 for thematic analysis. Thematic analysis allows themes from different levels in the data to surface. An important part of this qualitative method of analysis is to devise a coding framework that helps to structure and reveal themes in a text [24]. This coding framework contains various levels and is constructed on the basis of the theoretical framework (i.e. model of Rhee [20]) guiding the research question as well as on salient issues that arise in the text. Two trained researchers independently analyzed the data, and any discrepancies were discussed until total agreement was reached.

## Results

The three broad parental dimensions of Rhee [20] (i.e. global influences, general behaviors and specific feeding practices) were used as a framework to categorize the aspects that were mentioned by the three groups of caregivers. We will only report strategies that were mentioned by all three groups. Key quotes per group of caregivers about *effective* and *feasible* strategies to promote healthy eating in young children are included in the corresponding results section; additional examples are shown in Table 3.

**Table 3:** Quotes from focus group discussions

Categories	Parents	Family child care providers	Daycare assistants
Specific feeding practices			
- Rewarding	“When they know there will be dessert, they are always very eager to clear their plate”	“She didn’t eat soup, potatoes or fruit. We started with little portions. If she had eaten three spoons, she would get a big applause. Now, 8 months down the line, she clears her plate”	“Children know that when they clear their plate, they get their dessert. This stimulates them to eat. However, I read somewhere that by rewarding, you give children a sign that the food is not good.”
- Verbal encouragement	“I motivate and encourage my child to taste all sorts of vegetables, and that works.” “If my daughter does not like something, I say: Come on, let’s taste it”	“It depends on how you present it to them: motivating and encouraging children makes them want to eat”.	You need to encourage some children more than others: “Go ahead, you can eat it.”
- Rules	“He has to taste; that is not a point of discussion anymore”	“I do force them to taste at least once”	“They always have to taste at least once”
General behaviors			
- Sensory sensations			
Odor and taste	“The delicious smell of food makes children want to taste and eat.” “Taste, food needs to have a good taste”.	“If I prepare something, then I taste to check if it tastes good. Otherwise I should not expect the children to like it.”	“The food has to smell good. That makes children want to taste and eat.”
Visual			
Presentation of the food	“Sometimes, I make faces with the food. In the meantime, they are laughing and are distracted, and they don’t realize they are eating.”	“A lot of children do not like mixed foods. Especially picky eaters do not want to eat porridge. By offering the ingredient in its entirety, I convinced a few children to eat; children who were not willing to eat at home.” “I sometimes make a ‘fruitcake’: I arrange pieces of fruit in the form of a decorated cake. Then, they are very motivated to eat the fruit.”	“We won’t put disliked vegetables on their plate together with the other foods. We place them on a separate plate. If these disliked vegetables are on their plate, and the juice of these vegetables is all over their plate, mixed with the other foods, they do not want to eat anymore.”
Table layout	“We have one special playful plate, and if my child does not like something, they get that plate. Then they do like it”	“A plate or cutlery with a figure helps them to eat and taste”	“The color of the cutlery”
- Involvement	“He likes to help in the kitchen. When he helps cooking, it is much easier for him to eat it.”	“When they can help in the kitchen, that is a good motivation to taste: by helping, they are tasting along the way”	“Involving them in the process of cooking would really help”
- Variation	“My son has difficulties with textures. If I vary enough, he still wants to keep trying.”	“Give them a variety of food while they still eat everything. I vary a lot, and that helps for their eating later on.”	“Variation makes children curious. If you always give potatoes and sausages, then they only want to eat that. Variation is really necessary, I think”
- Modeling	“If a parent does not eat a wide variety of food, the children won’t either.”	“When they see that other children are eating, they are motivated to eat themselves.”	“The daycare assistants are asked to taste, to stimulate the children to taste”
- Repeated exposure	I tell them it can take up to 25 times before they will like it. Then I say: “Come on, only 25 times to go” … “only 24 …” and that works.	“Don’t think that they don’t like it when they don’t eat it the first time. Keep on presenting it to them, and eventually, they will like it”.	“If they don’t like it at first, keep on presenting it to them, several times”
Global influences			
- Atmosphere	“We talk and laugh, and in the meantime they are eating”	“A peaceful atmosphere is very important. If you are calm yourself and do not have too much stress, they’re going taste and eat better.”	“We try to create a cozy, homelike situation”

### Specific feeding practices

#### Rewarding

The first strategy that participants used to effectively influence children’s eating behavior was rewarding. There were considerable differences in the type of reward that was given and the behavior that was rewarded. Some participants used pleasant activities as a reward, while others resorted to desserts. Some rewarded clearing the plate, while others rewarded tasting behavior. There seemed to be no consistency within or between groups of caregivers in this respect. Although daycare assistants seemed to reward children less than the other groups, no practical barriers were mentioned regarding the applicability of this strategy.

#### Verbal encouragement

Another strategy that was mentioned was verbal encouragement. As was the case with rewarding, some differences were found in the kind of behavior that was encouraged. Again, while some caregivers encouraged clearing the plate, others encouraged tasting behavior, and there was no apparent consistency within or between groups of caregivers. Here too, no barriers or problems were mentioned regarding the applicability of the strategy.

#### Rules

Most of the caregivers in the three different groups applied the rule that children had to taste the given food at least once. It seemed crucial for most of them that their children tasted all food items, as is illustrated by the following quote:

*“I force them to taste. Persistence pays off”. (Family child care provider)*

However, no rationale was offered for why they found this so important. Again, no barriers or problems were mentioned regarding the applicability of the strategy.

### General behaviors

#### Sensory sensations

##### Smell and taste

The three groups of caregivers acknowledged the importance of attractive sensory sensations of food, meaning that the food had to smell and taste good. Some family child care providers and most daycare assistants expressed that this aspect is unfortunately beyond their control. In daycare centers, the rules about hygiene and safety have become very rigid, meaning that their budget forces them to offer food from an institutional kitchen, which is perceived as less tasty than food what they would be able to prepare themselves in the daycare center.

*“Unfortunately, a catering company is much cheaper than hiring a cook and adapting the kitchen to the rigid rules.” (Daycare assistant)*

In contrast, family child care providers cook at home. However, they receive a fixed amount of money per child. If they choose to cook fresh and healthy food – which tends to be more expensive than canned food – they do not get more money. As a result, they feel somewhat restricted in their efforts to offer healthy, tasty food that smells good.

*“We earn 15 euros per child, and I try to buy fresh products as much as possible, but I do not get extra money […]. It is possible if you only have a few children, but if you have more, it becomes financially much more difficult.” (Family child care provider)*

##### Visual aspects

Besides smell and taste, a third form of sensory sensation that was mentioned was the visual aspect. Visual stimuli can be subdivided into (a) how the food itself is presented and (b) how the meal is “framed” (i.e. table layout: the type of plate, the color of the cutlery). Despite some caregivers not being able to fully control how the food smells and tastes, they can, up to a certain level, control how it is presented. They all agreed on the importance of a visually attractive meal to make children taste and eat their food.

*“Children eat with their eyes” (Parent; Family child care provider)*

Nevertheless, they all had their own ideas on which food presentations make children taste and eat healthy food. The caregivers’ opinions seemed to differ individually rather than per group. For example, regarding how the food itself is presented, some claimed that picky eaters are not eager to eat mixed foods, since they do not know what is inside. Therefore, offering an ingredient in its entirety motivates children to eat. Others claimed that mixed food lowers the threshold to eat. Although daycare assistants have less control over how the ingredients are presented (i.e. most daycares offer food that is prepared in an institutional kitchen), they still find their own ways to present the food as attractive as possible (e.g. placing disliked vegetables on a separate plate, see Table 3). Caregivers did also influence the way food is presented to children in a different way, for example by using colorful cutlery or playful plates. Although the importance of a visually attractive meal is acknowledged by all caregivers, daycare assistants seemed to apply this kind of child-friendly framing less than the other groups of caregivers.

#### Involvement

The three groups of caregivers highlighted the importance of children’s involvement in cooking (e.g. through observation or by helping) and cooking-related activities (e.g. by having a kitchen garden). However, in practice, this is much more feasible for parents than for daycare assistants and family child care providers. Due to rigid hygiene and safety rules, daycare assistants do not prepare any meals themselves and are not even allowed to make a healthy afternoon snack, like a fruit salad. As a consequence, they cannot involve the children in preparing the food.

*“The children saw us while we were peeling apples. They were very curious. Unfortunately, due to hygiene rules, we are not allowed to do that anymore.” (Daycare assistant)*

In spite of these restrictions, they still find their own ways to involve the children as much as possible in food-related activities.

*“Every day we have a kitchen prince or princesses, who is allowed to put the apples in the dish.” (Daycare assistant)*

*“We used to fill the children’s plates in the room next to where they were sitting. But now, we fill the plates on the table in front of them. That way, they are a little more involved”. (Daycare assistant)*

To summarize, children in daycare centers cannot observe the cooking or help with the cooking. In contrast, children with family child care providers *can* often do this as most family child care providers cook at home.

*“As soon as I start cooking, they keep an eye on me, and they don’t go away”. (Family child care provider)*

One major difficulty that family child care providers experience when they involve children in preparing the food is that they are on their own. Moreover, the child to caregiver ratio is often higher in family child care homes than in daycares. Most of the family child care providers have at some point tried an activity in which everyone prepared food together. For most of them, that was not a positive experience at all and rather messy.

*“Once, we did a cooking activity with the children. We had only begun, and their clothes were already smeared with food.” (Family child care provider)*

As a result, family child care providers avoid cooking together but try to involve the children in simpler things, for example stirring the soup or picking vegetables from the kitchen garden.

#### Variation

The caregivers in the three different groups pointed out that offering a variety of food has a positive influence on different aspects of children’s eating behavior. Again, this is beyond the control of most daycare assistants, since they do not decide what food is offered to the children. Nevertheless, they were moderately satisfied with the variety of food available for *toddlers*. The baby food, however, is all canned and all tastes the same and they regret that they can no longer prepare vegetable porridge themselves.

*“We are not allowed anymore to make vegetable porridge for the babies. Now, we offer them canned foods. But we are not at all satisfied with this change. They all taste the same. […] It is also contradictory, as a baby, they need to eat canned food, and suddenly, when they grow up, they need to eat fruit, and they don’t know that.” (Daycare assistant)*

*“It’s a big difference, because it is a unilateral taste you offer to the children.” (Daycare assistant)*

All three groups of caregivers were convinced that offering a variety of food is very important for taste development. Up to a certain level, parents and family child care providers can control this and make efforts to offer a variety of food.

#### Modeling

The three groups of caregivers noted that a role model has a positive influence on children’s eating behavior. Two types were mentioned. The first role model that can motivate children to taste food are peers (i.e. the other children). However, peer modeling during mealtime is not always feasible, especially for family child care providers and daycare assistants, who often have several babies to look after. As babies eat and sleep at their own pace and do not sit at the same table, peer modeling cannot be applied here. For the older children, this *can* be a factor as they do eat together and can therefore influence each other. The second type of role model are adults. Family child care providers are aware that they can act as a role model and encourage children to eat. However, in their opinion, modeling during mealtime is not feasible for very practical reasons, as they are constantly busy feeding the children and therefore do not have the time to sit and eat with the children.

*“The ideal image is that we all eat together, but in practice, if you have a lot of children, that’s very difficult.” (Family child care provider)*

Similarly, daycare assistants have the intention to sit and taste together with the children, but in real life, that does not always happen.

*“We are asked to accompany the children at the table. […] However, you’ll see that it is not always how we want it to be: daycare assistants walk around instead of sitting next to the children. Sometimes it is beyond our control: if a daycare assistant is ill, they do not get replaced and we have more work.” (Daycare assistant)*

Parents are also convinced that modeling is important, and they try to eat together as much as possible, to act as a role model. Similar to family child care providers and daycare assistants, this is not always possible due to their busy schedules, though (see “atmosphere”).

#### Repeated exposure

Most of the caregivers also stated that you have to repeatedly expose a child to a vegetable that it does not like. Only one family child care provider and one parent did not agree with this idea.

*“If they do not like it, I won’t offer it anymore.” (Parent)*

As mentioned above, parents and most family child care providers can control what they offer their children. Since this is not the case for daycare assistants, they cannot choose to repeatedly expose the children to a vegetable they do not like on specified moments (e.g. every three days). They can, however, control what they ask children to do when they offer them a disliked vegetable (i.e. asking them to try one bite).

### Global influences

#### Atmosphere

Caregivers highlighted the importance of a cozy, light-hearted and peaceful atmosphere during mealtime. In this kind of atmosphere, children are more likely to taste and eat. The explanation of the caregivers is that in this stress-free setting everyone will be talking about everyday events, which distracts children in a positive way and gets them to eat without being fully aware of it. Creating a peaceful atmosphere is often a challenge for family child care providers and daycare assistants, since they have more children than a regular family. Still, they try to create a cozy, homelike situation, for example, by getting the children to eat in little groups.

*“In our daycare center, we eat in little groups of maximum five children, to create a homey atmosphere.” (Daycare assistant)*

For most parents, this seems to be particularly challenging in the morning.

*“In the morning, everything needs to be ready in half an hour. If they don’t eat in that time, they have to go to school without food”. (Parent)*

For some parents, it is a challenge for all meals, due to their busy lives.

*“We almost never eat together anymore, not even on weekends. Only on Sundays can we eat together. Saturday it is football for the children. I do not like that, because the family feeling is gone”.*

## Discussion

The current study examined **(a)** what behaviors are, according to three different groups of caregivers (i.e. parents, family child care providers, daycare assistants), most effective in promoting healthy eating in young children and **(b)** whether or not these behaviors are feasible in their specific settings. These behaviors were categorized within the theoretical model of Rhee [20], in which (parental) influences on a child’s dietary habits are divided into three broad categories. They will be discussed below in the light of current scientific research on how to improve young children’s eating behavior.

### Specific feeding practices

Three behaviors – rewarding, verbally encouraging and imposing a taste-rule – could be categorized under “specific feeding practices”, since they addressed the child directly, with the intent to influence eating behaviors, such as tasting, eating and liking. The three groups of caregivers reported no problems concerning the feasibility of these behaviors in their specific setting. This can be explained by the nature of these specific feeding practices: they involve single and simple behaviors, independent of environmental constraints. The three practices have in common that they involve an instruction to perform a certain behavior and only differ in the sense of what follows after the behavior has been performed. In the case of rewarding, a reward is announced, which can be a non-tangible social reward or a tangible reward. We assume that verbal encouragement is in fact a kind of social reward since it implies that the caregiver will be proud if the behavior is carried out. In the case of imposing a taste-rule, the consequences were not specified by the caregivers.

Most of the caregivers used rewards or verbal encouragement to influence children’s eating behavior. However, they had different opinions concerning **(a)** the specific behavior they expect from the child (e.g. tasting behavior or clearing the plate) and **(b)** the type of reward (e.g. a dessert, a non-token reward). The use of rewards or verbal encouragement to facilitate desirable eating behavior can be conceptualized as operant or instrumental conditioning. This learning process implies that an individual's behavior is modified by its positive (i.e. reinforcement) or negative (i.e. punishment) consequences [25]. When a specific behavior has unpleasant consequences, the frequency of that behavior will decrease. Conversely, if a behavior is followed by a rewarding stimulus, this behavior will be repeated in the future. It has indeed been shown that rewards can have a positive influence on children’s eating behavior (for a review, see [26]), but only if used appropriately. For example, the type of *behavior being encouraged* plays an important part in whether or not positive effects will be shown. Encouraging children (verbally or with a reward) to clear their plate may undermine their internal regulation system and lead to overweight [27–29]. Conversely, encouraging children to taste a food item they dislike might eventually have positive effects on liking and consumption [30], but only if the child is not intrinsically motivated to taste. According to Social Determination Theory, an extrinsic motivator (i.e. a reward) undermines intrinsic motivation (for a review, see [31]). A second factor that determines the success of rewards is what *type of reward* is given. Offering sweets as a reward provokes negative effects, as it enhances children’s preference for sweets [32]. In contrast, various studies have demonstrated that both non-food tangible rewards (e.g. stickers) and non-tangible rewards (e.g. praise) enhance children’s liking and consumption of disliked food items [33–35]. These findings indicate that the effectiveness of rewards in the context of eating behavior is more complex than the general principle of operant conditioning, as formulated by Skinner [25]. Based on the different opinions and statements of all caregivers, we can infer that most of them are not aware of **(a)** what behavior to encourage (i.e. tasting vs how much a child ate) and **(b)** the importance of considering the type of reward.

The last specific feeding practice put forward was to impose a taste-rule. All three groups agreed that children had to taste at least once a meal. This rule can lead to familiarization and eventually liking and increased consumption of the vegetable [36]. However, it can also have counterproductive effects if accompanied by pressure and negativity [37]. Furthermore, the definition of “tasting” is often misunderstood among caregivers. Caregivers often expect their children to swallow the food item, which is usually the hardest part. The critical aspect of “tasting” in the process of liking a food item is familiarizing children with the taste by bringing the food item in contact with the taste buds. Obligating children to swallow may even decrease liking of the food item and increase a food neophobic reaction [38]. We could not infer from the focus group interviews that caregivers were aware of the specificity of the taste-rule or knew how to react when the child disobeyed the rule.

### General behavior

This category contains five aspects (i.e. sensory sensations, involvement, variation, modeling, and repeated exposure) that are not immediately directed toward the child, but do have an influence on the child’s eating behavior. The importance of these five behaviors was acknowledged by all three groups of caregivers. However, in contrast to the specific feeding practices, these general behaviors seemed far less feasible, depending on the type of caregiver. As the menu is beyond the control of daycare assistants and some of the family child care providers, they cannot choose to repeatedly expose children to a certain food, make sure that there is enough variation, or determine how the food tastes or smells. Obviously, they *can* choose how they present the food and whether or not to impose a taste-rule (see above). Involvement is the least feasible for daycares, since they have rigid hygiene and safety rules: the food is not prepared in the daycare center, so children cannot observe the cooking or be involved in the cooking process. Family child care providers face a different kind of problem: children can observe them while they are cooking, but it is difficult for them to involve the children, because they are usually on their own and have more children to look after than daycare assistants. For the same reason, adult modeling during mealtime is the least feasible for family child care providers. Although there are more employees in daycares, adult modeling during mealtime also seems difficult for daycare assistants. In contrast to the United States, where federal standards help to ensure that caregivers in childcare settings (e.g. Head Start programs) model healthy eating behavior by sitting with children during mealtime [39–41], no such standards exist for Belgian childcare settings. As a consequence, the request to sit and eat with the children might be seen as an option instead of a necessity. Peer modeling is the least feasible for family child care providers, since they have children of different ages, with babies eating on their own rhythm and thus at different moments. This is less the case for daycares, where children of the same age eat together in small groups, and can act as a model.

Research has shown that these general behaviors have a positive influence on children’s eating behavior [20, 36, 42, 43]. First of all, providing enough food variety and continuing to offer children all kinds of food (even if they insist that they do not like it) is beneficial for taste development and the acceptance of food [44]. Second, repeatedly exposing children to the taste of food items (i.e. Repeated Exposure) is proven to be effective in increasing children’s liking and consumption of vegetables that they initially disliked [36, 45, 46]. Next, according to Social Cognitive Theory [47], modeling can also be very influential in establishing learning and behavioral change. Not only adults (familiar as well as unfamiliar ones) seem to be effective role models [48–50], but also peers [42, 51], and even cartoon characters have a positive influence on children’s eating behavior [52]. Furthermore, we assume that attractive sensory sensations and involvement create a positive context in which healthy eating behavior may be facilitated. This can be explained by flavor-context learning, which is a form of classical conditioning. According to the principle of classical conditioning, an individual's behavior is modified as a consequence of repeated pairings of two stimuli. A stimulus that initially provokes no reaction (i.e. neutral stimulus) is paired with a meaningful biologically relevant stimulus (i.e. unconditioned stimulus) that automatically provokes a reaction (i.e. unconditioned response). After repeated pairings, the initially neutral stimulus starts to elicit the same response as the biologically relevant stimulus (i.e. conditioned response) [53, 54]. In the case of flavor-context learning, children associate food (i.e. neutral stimulus) with the emotional valence of the social context (i.e. unconditioned stimulus). The preference of food will increase (i.e. conditioned response), if the food is accompanied by positive behaviors or aspects [27], for example by attractive sensory sensations or by involving children in a positive way. Conversely, providing food in a negative social context (e.g. by means of coercive feeding techniques) will lead to a decrease in food preferences [37]. We could not infer from the focus group interviews that caregivers were aware of the “long-term” character of these general behaviors; since these behaviors imply a learning process, caregivers cannot expect to detect any immediate changes in tasting or liking behavior. If they continue carrying out these behaviors, then changes will occur eventually.

### Global influences

A peaceful, cozy atmosphere could be categorized under global influences, since it is a consequence of multiple behaviors and aspects (e.g. the above-mentioned general behaviors, the functioning of the group). Everybody agrees that a cozy, homelike situation is important, but it is challenging for the three groups of caregivers. A positive climate indeed facilitates healthy eating behavior [20], which can also be explained by flavor-context learning: the preference of food will increase, if the food is accompanied by a cozy atmosphere.

### Strengths, limitations and future research

The main strength of this study is the inclusion of different groups of caregivers in a Flemish population. As many young Flemish children spend a significant amount of time in child care [19], family child care providers and daycare assistants have considerable impact on children’s eating behavior. To our knowledge, little research has focused on how children’s eating behavior can be improved in the Flemish childcare system. In the United States, more research has been conducted on obesity prevention in childcare settings [9, 55–57]. However, due to cultural and policy differences, these findings and conclusions are not necessarily valid for a Flemish population. Since childhood obesity as well as unhealthy eating behaviors are rising in Flanders too, there is an urgent need for studies in this population.

A few limitations of this study need to be considered. First, for daycare assistants and family child care providers, we only studied one focus group per type of caregiver. Therefore, thematic saturation might not have been reached and valuable information might have been missed. For example, we could not infer from the focus groups whether or not children serving themselves was perceived as an effective strategy to promote healthy eating behavior. Furthermore, we may also question whether overall themes can actually be derived from this limited population. However, as all daycare assistants and family child care providers in Flanders follow the policy guidelines of the national institute “Child and Family” (i.e. Kind&Gezin), it can be assumed that the current findings are not unique to the sites of the participants. Nevertheless, more groups per type of caregiver could have strengthened the generalizability of the overall themes. Second, since daycare assistants do not have a lot of control over policies, including the menu, future research should address the authorities or individuals responsible for the menu as well as daycare directors for their attitudes, beliefs and policy concerning healthy food. Third, as the current study dealt with events just before and during mealtime, we may have missed behaviors during other times of the day, for example, when children see that a caregiver is eating fruit in the afternoon (i.e. adult modeling). Fourth, the recruitment procedure may have introduced a selection bias. Caregivers interested in healthy food were probably more inclined to participate, which may have been why they all were very much aware of the importance of a lot of the health-improving behaviors. Fifth, promoting healthy eating behavior is a complex and difficult matter which depends on multiple factors. For example, the three levels of Rhee [20] interact, and depending on the global influences (i.e. a good or bad atmosphere), the individual practices will work better or worse [20]. To grasp the interaction between these levels, individual in-depth interviews would be more appropriate. Finally, previous research has shown that the effectiveness of some strategies might depend on individual differences in child characteristics, such as reward sensitivity [58] or food responsiveness [59]. Besides general guidelines on how to improve healthy eating behavior, caregivers should be aware that children are individuals and that some may benefit more from strategy X than from strategy Y. Whether caregivers take these individual differences into account does not emerge from our data, but this could simply be because we did not specifically ask for it. This limitation should be addressed by future research.

### Implications

The current study provides first-hand information on caregivers’ perceived effectiveness and feasibility of strategies used at home, in daycare centers and in family child care homes to promote healthy eating in young children. We can infer from the focus group interviews that caregivers use various techniques to accomplish this. However, our findings also show that they often do not have any specific knowledge on how to apply a particular strategy. There is therefore a need for better training of caregivers [55], with specific evidence-based written or visualized material, in which clear and concrete guidelines clarify what strategies to apply, and how to apply them (e.g. when and how to reward behavior). A training program for child care providers could be based on existing evidence-based methods in the United States (i.e. Nutrition and Physical Activity Self-Assessment for Child Care, NAP SACC [60, 61]), which include facility-level interventions. The implementation of effective parent-led home-based interventions [62–64] could help parents to improve their children’s eating behavior. Research has shown that both a parent-led home-based intervention consisting of specific information on repeated daily tastings of a vegetable [64], and additional instructions on modeling and rewarding [62] effectively increased children’s liking and consumption of a disliked vegetable, while such effects were not obtained from nutritional advice or leaflets [64]. Furthermore, our results show that strategies vary in applicability. For example, caregivers expressed the difficulty of eating together with the children which could facilitate modeling. If this perceived difficulty is caused by policy-makers not attaching enough weight to this strategy, more efforts are needed to convince all stakeholders of the importance of modeling. If necessary, certain rules should be tightened to solve this problem (e.g. in daycares, the assistants should be obliged to sit and eat with the children). However, if it appears that more stringent rules do not produce the desired effect due to workload, a rotation system could be developed or, ideally, more staff should be hired. In other cases, reforms should be applied. For example, the problem of children eating on their own rhythm, which hinders peer and adult modeling, can be solved by grouping together children of the same age. Furthermore, the current restrictions imposed by the authorities (e.g. it is not allowed to prepare food in the daycare center) are justifiable for the sake of hygiene and safety, but interfere with many possibilities to pursue a policy promoting healthy eating behavior. A compromise should be reached to create a hygienic and safe environment in which healthy food is promoted as well. For example, the food should be prepared in a place that can be observed safely by all children at any time. On some days, older children should be allowed to help with simple and safe tasks, such as stirring the soup, washing vegetables or cutting vegetables with a blunt knife. Rules such as washing hands before entering the kitchen, storing food at the right temperature, and cleaning kitchen equipment may in turn contribute to hygiene. In the United States, the systematic QRIS approach (i.e. Quality Rating and Improvement Systems [65]) has been used to assess, improve and communicate the level of quality in early care and education programs [66]. Specific policy implementation strategies (i.e. professional development, assessment, technical assistance, and incentives) are recommended to improve eating, physical activity and breastfeeding practices and limit screen time in child care settings [67]. Examples of these recommendations that could also follow from the current results are including health as a mandatory policy element to reflect its importance in children’s development, or offering the advice of a consultant who could help address issues such as staff modeling behaviors [67].

## Conclusion

The three groups of caregivers largely agree on how healthy eating behavior should be promoted in children. Most of the behaviors and strategies mentioned are consistent with research on promoting healthy eating in young children. However, this is not the case for rewarding and verbal encouragement, since the caregivers all expressed different ideas about this topic, sometimes contradictory to research and the best accepted practices involved in positively influencing children’s eating behavior. This clearly demonstrates the need for better training for caregivers. Furthermore, it was found that the applicability of health-promoting behaviors largely depends on the caregiving setting (e.g. infrastructure, policy). Based on the current results, if policies concerning the setting remain unchanged, a general intervention that can be applied to all groups of caregivers should focus on specific feeding practices, since these involve simple behaviors that are not hindered by limitations of the caregiving setting. However, as the current study design did not allow us to qualitatively compare the effectiveness of the different practices (e.g. are specific feeding practices more effective than general behaviors?), further research is needed to strengthen these recommendations.

## Additional file

Additional file 1: Consolidated criteria for reporting qualitative studies (COREQ): 32-item checklist. (DOCX 20 kb)

## Abbreviations

VBJK: Flemish Centre for Innovation in the Early Years
COREQ: Consolidated criteria for reporting qualitative research
