# Erratum to: Perceived effective and feasible strategies to promote healthy eating in young children: focus groups with parents, family child care providers and daycare assistants

**DOI:** 10.1186/s12889-017-4092-3

**Published:** 2017-02-10

**Authors:** Laura Vandeweghe, Ellen Moens, Caroline Braet, Wendy Van Lippevelde, Leentje Vervoort, Sandra Verbeken

**Affiliations:** 1 Department of Developmental, Personality and Social Psychology, Ghent University, Henri Dunantlaan 2, 9000 Ghent, Belgium; 2 Department of Public Health, Ghent University, De Pintelaan 185, 9000 Ghent, Belgium

## Erratum

Upon publication of this article [1], it was brought to our attention that Table 3 was incorrectly presented. The correct Table 3 is shown below:

**Table 3:** Quotes from focus group discussions

Categories	Parents	Family child care providers	Daycare assistants
**Specific Feeding Practices**			
- Rewarding	“When they know there will be dessert, they are always very eager to clear their plate”	“She didn’t eat soup, potatoes or fruit. We started with little portions. If she had eaten three spoons, she would get a big applause. Now, 8 months down the line, she clears her plate”	“Children know that when they clear their plate, they get their dessert. This stimulates them to eat. However, I read somewhere that by rewarding, you give children a sign that the food is not good.”
- Verbal encouragement	“I motivate and encourage my child to taste all sorts of vegetables, and that works.” “If my daughter does not like something, I say: Come on, let’s taste it”	“It depends on how you present it to them: motivating and encouraging children makes them want to eat”.	You need to encourage some children more than others: “Go ahead, you can eat it.”
- Rules	“He has to taste; that is not a point of discussion anymore”	“I do force them to taste at least once”	“They always have to taste at least once”
**General Behaviors**			
- Sensory Sensations			
Odor and Taste	“The delicious smell of food makes children want to taste and eat.” “Taste, food needs to have a good taste”.	“If I prepare something, then I taste to check if it tastes good. Otherwise I should not expect the children to like it.”	“The food has to smell good. That makes children want to taste and eat.”
Visual			
Presentation of the food	“Sometimes, I make faces with the food. In the meantime, they are laughing and are distracted, and they don’t realize they are eating.”	“A lot of children do not like mixed foods. Especially picky eaters do not want to eat porridge. By offering the ingredient in its entirety, I convinced a few children to eat; children who were not willing to eat at home.” “I sometimes make a ‘fruitcake’: I arrange pieces of fruit in the form of a decorated cake. Then, they are very motivated to eat the fruit.”	“We won’t put disliked vegetables on their plate together with the other foods. We place them on a separate plate. If these disliked vegetables are on their plate, and the juice of these vegetables is all over their plate, mixed with the other foods, they do not want to eat anymore.”
Table layout	“We have one special playful plate, and if my child does not like something, they get that plate. Then they do like it”	“A plate or cutlery with a figure helps them to eat and taste”	“The color of the cutlery”
- Involvement	“He likes to help in the kitchen. When he helps cooking, it is much easier for him to eat it.”	“When they can help in the kitchen, that is a good motivation to taste: by helping, they are tasting along the way”	“Involving them in the process of cooking would really help”
- Variation	“My son has difficulties with textures. If I vary enough, he still wants to keep trying.”	“Give them a variety of food while they still eat everything. I vary a lot, and that helps for their eating later on.”	“Variation makes children curious. If you always give potatoes and sausages, then they only want to eat that. Variation is really necessary, I think”
- Modeling	“If a parent does not eat a wide variety of food, the children won’t either.”	“When they see that other children are eating, they are motivated to eat themselves.”	“The daycare assistants are asked to taste, to stimulate the children to taste”
- Repeated Exposure	I tell them it can take up to 25 times before they will like it. Then I say: “Come on, only 25 times to go” … “only 24 …” and that works.	“Don’t think that they don’t like it when they don’t eat it the first time. Keep on presenting it to them, and eventually, they will like it”.	“If they don’t like it at first, keep on presenting it to them, several times”
**Global Influences**			
- Atmosphere	“We talk and laugh, and in the meantime they are eating”	“A peaceful atmosphere is very important. If you are calm yourself and do not have too much stress, they're going taste and eat better.”	“We try to create a cozy, homelike situation”
