# Supplementary material for: Perceived effective and feasible strategies to promote healthy eating in young children: focus groups with parents, family child care providers and daycare assistants
Source: BMC Public Health. 2016 Oct 4;16:1045. doi: 10.1186/s12889-016-3710-9 (PMC5050921; doi:10.1186/s12889-016-3710-9)
Supplement: Additional file 1: — Consolidated criteria for reporting qualitative studies (COREQ): 32-item checklist. (DOCX 20 kb) [file 12889_2016_3710_MOESM1_ESM.docx]

| Consolidated criteria for reporting qualitative studies (COREQ): 32-item checklist [1] | |
| --- | --- |
| 1. Interviewer/facilitator | The focus group interviews were conducted by Caroline Boudry and Wendy De Weyer |
| 1. Credentials | PhD’s, masters, and one master student |
| 1. Occupation | All authors are researchers at Ghent University. Caroline Boudry and Wendy De Weyer are researchers at the Flemish Centre for Innovation in the Early Years (VBJK) |
| 1. Gender | All female |
| 1. Experience and Training | The focus groups were led by two VBJK researchers who are experienced in conducting focus groups. A protocol was followed. |
| 1. Relationship established | Prior to study commencement, the interviewers and the participants had earlier professional contacts, but there was no conflict of interest (e.g. participants do not depend on funding of VBJK) that could have biased the answers of the participants. |
| 1. Participant knowledge of the interviewer | The participants know VBJK as a supporting team in developing quality childcare. |
| 1. Interviewer characteristics | Both interviewers work at VBJK. Since this research center is in close contact with a variety of caregivers, it was able to convince people who normally would not volunteer for group discussions. |
| 1. Methodological orientation and Theory | Content analysis, which brings themes at different levels in the data to the surface. The coding framework consists of different levels and is constructed on the basis of the theoretical model of Rhee [2] as well as salient issues that arise in the text. |
| 1. Sample | The participants were recruited by VBJK. Since this research center is in close contact with a variety of caregivers, it was able to recruit a purposive sample and convince people who normally would not volunteer for group discussions. |
| 1. Method of approach | The participants were recruited by VBJK through personal contacts (i.e. face-to-face and email). |
| 1. Sample Size | 4 focus groups were conducted (n = 33): two focus groups consisted of parents (n = 7 for each focus group), one focus group of family child care providers (n = 9) and one focus group of daycare assistants (n = 10) |
| 1. Non-participation | We have no details on non-participation |
| 1. Setting of Data Collection | The four focus groups were conducted at VBJK in a comfortable and neutral room |
| 1. Presence of non-participants | No |
| 1. Description of the sample | Mean age of all participants is 33.33 (*SD*: 5.40). Age and gender per type of caregiver is reported in table 1. |
| 1. Interview guide | A semi-structured questioning guide was developed by VBJK and reviewed by researchers from Ghent University. Questions are provided in table 2. It was not pilot tested. |
| 1. Repeat interviews | We did not carry out repeat interviews because this is not necessary for our research purposes. Repeat interviews are relevant in vulnerable populations and appropriate to capture something over time [3] . |
| 1. Audio-/visual recording | We used audio recording to collect the data |
| 1. Field notes | During the interview, field notes were made |
| 1. Duration | The discussions lasted approximately 90 minutes |
| 1. Data Saturation | The topic of data saturation is discussed in the article |
| 1. Transcripts returned | No |
| 1. Number of data coders | 2 |
| 1. Description of the coding tree | Yes, the three broad parental dimensions of Rhee [2](i.e. global influences, general behaviors and specific feeding practices) were used as a framework to categorize the aspects that were mentioned by the three groups of caregivers. The coding three is described in the article, but not shown in a figure. |
| 1. Derivation of themes | Themes were derived from the data as well as from the theoretical model of Rhee [2] |
| 1. Software | NVivo 10.0 |
| 1. Participant checking | No |
| 1. Quotations presented | Yes, participant quotations are presented to illustrate the themes. It is made clear from which type of caregiver each quotation originated. |
| 1. Data en findings consistent | Yes |
| 1. Clarity of major themes | Yes |
| 1. Clarity of minor themes | If a theme is less present in a type of caregiver, it is mentioned. |

1. Tong, A., P. Sainsbury, and J. Craig, *Consolidated criteria for reporting qualitative research (COREQ): a 32-item checklist for interviews and focus groups.* International Journal for Quality in Health Care, 2007. **19**(6): p. 349-357.

2. Rhee, K., *Childhood overweight and the relationship between parent behaviors, parenting style, and family functioning.* The ANNALS of the American Academy of Political and Social Science, 2008. **615**(1): p. 11-37.

3. Vincent, K.A., *The advantages of repeat interviews in a study with pregnant schoolgirls and schoolgirl mothers: piecing together the jigsaw.* International Journal of Research & Method in Education, 2013. **36**(4): p. 341-354.
